# Valorization of Sugarcane By-Products through Synthesis of Biogenic Amorphous Silica Microspheres for Sustainable Cosmetics

**DOI:** 10.3390/nano12234201

**Published:** 2022-11-26

**Authors:** Joana R. Costa, Ana Paula Capeto, Carla F. Pereira, Sílvia S. Pedrosa, Inês F. Mota, João da Silva Burgal, Ana I. Pintado, Manuela E. Pintado, Catarina S. S. Oliveira, Patrícia Costa, Ana Raquel Madureira

**Affiliations:** CBQF—Centro de Biotecnologia e Química Fina—Laboratório Associado, Escola Superior de Biotecnologia, Universidade Católica Portuguesa, Rua de Diogo Botelho 1327, 4169-005 Porto, Portugal

**Keywords:** circular economy, amorphous silica, spherical microparticles, cosmetic ingredient, process development

## Abstract

Ashes from sugarcane by-product incineration were used to synthesize silica powders through alkaline hot extraction, followed by ethanol/acid precipitation or the sol–gel method. Both production methods allowed amorphous spherical silica microparticles with sizes ranging from 1–15 μm and 97% purity to be obtained. Water absorption ranged from 135–155 mL/100 g and 150–250 mL/100 g for precipitated silica and silica gel, respectively, while oil absorption ranged from 305 to 390 and from 250 to 350 mL/100 g. The precipitation with ethanol allowed the recovery of 178 g silica/kg ash, with a lab process cost of EUR 28.95/kg, while the sol-gel process showed a yield of 198 g silica/kg ash with a cost of EUR 10.89/kg. The experimental data suggest that ash from sugarcane by-products is a promising source to be converted into a competitive value-added product, minimizing the environmental impact of disposal problems.

## 1. Introduction

In Brazil and India, the sugarcane industry generates more than 800 million metric tons of sugarcane waste per year, particularly straw and bagasse [1]. This waste is mostly burned as fuel in boilers to produce the water vapor necessary to produce sugar and ethanol and in energy cogeneration processes, resulting in the generation of 3–12 million tons of sugarcane ash/year [2,3]. This co-product is easily overlooked in landfills and commonly used as a fertilizer, although this is not a proper application due to its high silica content and lack of nutrients [3].

Sugarcane ash has high silica (SiO_2_) content, above 60 g/100 g ash, in crystalline and amorphous phases, such as quartz and cristobalite. The primary source of silica is the silicic acid available in the soil, which is absorbed by the sugarcane roots and transported to the leaves, where it is deposited as amorphous silica [4]. A significant amount of crystalline silica, mainly quartz, is also present in sugarcane ash derived from the sand stuck to the plant during the growing and harvesting processes. Nevertheless, part of the amorphous silica present in ash can be converted into crystalline form during uncontrolled combustion furnace conditions [4].

Silica is considered a value-added product for different industries, and its application is dependent on the morphology, size, solubility, and porosity of particles. Although several works have focused on the production of silica from biomass ashes, particularly from rice husk and sugarcane fly ashes, these materials are commonly assigned to material applications [5,6,7,8]. Methods for extraction of amorphous silica from sugarcane bagasse ash mostly include acidic pretreatment (mostly with HCl) followed by alkaline extraction with NaOH at 95 ± 5 °C and a precipitation step with HCl to neutralize the solution [9,10,11]. These methods allow high-purity silicas (99%) to be obtained, although they lack a finalization step that allows spherical particles suitable for cosmetics to be obtained.

In cosmetics, silica has a wide range of applications, such as absorbent, anti-caking, conditioning agent, non-surfactant, rheological modifier, and bulking agent applications, and it can also function as a microplastic alternative in exfoliating products [12].

In the past decade, increased demand has been observed for natural and sustainable skincare products [13]. The Statista Data Platform reported an expected increase in the global market value for natural cosmetics and personal care products from USD 34.5 billion in 2018 to approximately USD 54.5 billion in 2027, indicating that up to 52% of consumers prioritize natural, vegan, and sustainable beauty products [14].

This study aimed to provide a valorization pathway of an abundant agro-industrial by-product, sugarcane bagasse ashes, through the synthesis of biogenic silica prototypes for potential application in the development of more sustainable cosmetics.

## 2. Materials and Methods

### 2.1. Materials

Sugarcane bagasse ash (SCBA), obtained through the incineration of sugarcane bagasse, was provided by Raízen (Brotas, São Paulo, Brazil). The raw material was sieved, and the fraction with a particle size above 160 μm was used for the extraction of biogenic silica. Absolute ethanol (p.a > 99.8%) and sulfuric acid (p.a 95.0–97.0%) were purchased from Honeywell (Charlotte, NC, USA), and sodium hydroxide pellets were purchased from LabChem (Zelienople, PA, USA). Nitric acid 65% (Suprapur) and fluoric acid 40% were acquired from Merck KGaA (Darmstadt, Germany). Commercial silica Spheron L-1500 was a gift from Presperse (Somerset, NJ, USA).

### 2.2. Characterization of SCBA

SCBA was characterized for total ashes, moisture content, and total organic matter following the Association of Official Analytical Chemists (AOAC) methods [15,16].

Silica and other mineral contents were determined by Inductively Coupled Plasma–Atomic Emission Spectrometry (ICP–AES). Briefly, 200 mg of SCBA were mixed in a Teflon vessel with 7 mL of HNO_3_ 65% and 2 mL of HF 40% and heated in a microwave system (Berghof, Eningen, Germany). The digestion procedure was conducted in three steps: 140 °C and 40 bar for 5 min; 160 °C and 40 bar for 10 min; 200 °C and 40 bar for 30 min; and 100 °C and 20 bar for 5 min. After microwave digestion, the samples were cooled down to room temperature and diluted to a final volume of 50 mL. Microwave-digested samples were then analyzed through ICP–AES (PerkinElmer, Waltham, MA, USA) and quantified through external standard calibration. All the analyses were performed in triplicate.

### 2.3. Production of Biogenic Silica Microparticles

#### 2.3.1. Production of Na_2_SiO_3_ from SCBA

Nickel-plated stainless steel crucibles were used to mix ash and sodium hydroxide (SCBA: NaOH pellets mass ratio of 1:2), and the mixture was placed into a muffle oven at 350 °C for 20 min. After the removal of the crucibles, deionized water was cautiously added (mass ratio SCBA: H_2_O of 1:10), and the mixture was thoroughly stirred and filtered through a paper filter. The solid fraction was discarded, and the liquid fraction, rich in sodium silicate, was stored at room temperature.

#### 2.3.2. Production of Silica Gel Microparticles

For the production of silica gel microparticles, the pH of Na_2_SiO_3_ solution was adjusted to 6 using H_2_SO_4_ 10% (*v*/*v*) under agitation. The solution was then filtered under vacuum (pressure 300–400 mbar) at room temperature using a cloth filter and washed with deionized water using a mass ratio of gel solution: water of 1:3. The final gel was resuspended in water, the viscosity was adjusted to 10–12 cP using a Vibro Viscometer SV-10 (A&D Co., Ltd., Tokyo, Japan), and the solution was fed into a Niro Atomizer Mobile Minor Spray-Dryer using an inlet temperature of 180 °C and a pump flow of 2.5 L/h.

#### 2.3.3. Production of Precipitated Silica Microparticles

For the production of precipitated silica microparticles, absolute ethanol was added to the Na_2_SiO_3_ solution (volume ratio 1:1), and the pH was adjusted to 8.5 using H_2_SO_4_ 10% (*v*/*v*) under agitation. The solution was then filtered under vacuum (pressure 300–400 mbar) at room temperature using a cloth filter and washed with deionized water using a mass ratio of gel solution: water of 1:3. The final gel was dried overnight at 105 °C. The powder was ground, resuspended in deionized water at 20% (*w*/*w*), and stirred overnight at 50 °C. The final solution was fed into a Buchi B-191 Mini spray dryer. The operating conditions were as follows: inlet temperature, 160 °C; outlet temperature, 100–110 °C; gas flow, 600 L/h; pump rate, 0.23 L/h; aspirator rate, 25.5 m^3^/h.

### 2.4. Characterization of Silica Microparticles

#### 2.4.1. Chemical Composition

Briefly, 200 mg of biogenic silica were mixed in a Teflon vessel with 2 mL of HNO_3_ 65% and 2 mL of HF 40% and heated in a microwave system (Berghof, Eningen, Germany). The digestion procedure was conducted at 185 °C and 35 bar for 70 min. Microwave-digested samples were then analyzed through ICP–AES (PerkinElmer, MA, USA) and quantified through internal standard calibration. All analyses were performed in triplicate.

#### 2.4.2. Powder X-ray Diffraction

Powder X-ray Diffraction Analysis (PXRD) was performed on a Rigaku MiniFlex 600 diffractometer with Cu kα radiation with a voltage of 40 kV and a current of 15 mA (3° ≤ 2θ ≥ 60°; step of 0.01 and a speed rate of 2.0°/min).

#### 2.4.3. Fourier Transform Infrared Spectroscopy

Attenuated Total Reflection–Fourier Transform Infrared Spectroscopy (ATR–FTIR) analysis was conducted using a Spectrum 100 FTIR spectrometer (Perkin Elmer, Waltham, MA, USA) equipped with an ATR accessory (PIKE Technologies, Fitchburg, WI, USA) with a diamond/Se crystal plate. The number of scans was set to 64, and spectra were collected in the absorbance range of 4000 to 400 cm^−1^ with a resolution of 1 cm^−1^.

#### 2.4.4. Morphology

SEM microparticle analyses were performed on a JEOL-5600 LV Scanning Electron Microscope (Tokyo, Japan) from JEOL, Japan, equipped with a SPRITE HR Four Axis Stage controller (Deben Research, Suffolk, UK). Samples were placed on metallic stubs covered with adhesive carbon tape (NEM tape, from Nisshin, Japan) and coated with gold/palladium using a Sputter Coater (Polaron, from Bad Schwalbach, Germany). All observations were performed under high vacuum with an acceleration voltage of 30 kV at a working distance of 12–13 mm and a spot size of 20.

#### 2.4.5. Particle Size Distribution

Particle size distribution was analyzed in a Coulter LS 230 laser granulometer (Beckman Coulter, Pasadena, CA, USA) equipped with a small volume module. The measurement was made in water, using the Fraunhofer laser diffraction method. All the analyses were performed in duplicate. The particle size distribution (D_10_, D_50_, and D_90_) was size at which 10, 50, and 90% of the particles were under the average particle size and span (SPAN = (D_90_ − D_10_)/D_50_).

### 2.5. Oil and Water Absorption Capacity

Oil and water absorption capacities were determined on the basis of the method described by Isah et al. [17]. Briefly, one gram of precipitated or silica gel was weighed and suspended in 10 mL of deionized water or sunflower oil for water or oil absorption capacity, respectively. The sample was incubated at 60 °C for 30 min, followed by centrifugation at 422.2 rcf for 15 min. The supernatant was rejected, and the pellet was weighed again. Water and oil absorption capacities were calculated according to Equation (1):

(1) $$\frac{\mathrm{water}}{\mathrm{oil}}\mathrm{absorption}\;\left(\frac{\mathrm{mL}}{100\;g}\right)=\frac{\mathrm{final}\;\mathrm{mass}-\mathrm{initial}\;\mathrm{mass}}{\mathrm{initial}\;\mathrm{mass}}\times 100$$

### 2.6. Production Cost Estimation

The estimation of silica gel and precipitated silica production costs was based on the determination of chemical and energy costs plus overheads, which are considered 80% of the chemical and energy costs accounting for waste management, equipment depreciation, labor costs, and others [18]. A list of the key assumptions used to determine the production process costs is displayed, and all values are presented in euros.

Both processes were simulated on the basis of a target processing of 30.21 kg SCBA/day. The costs with reagents were determined according to Table 1 and the experimental mass balance (data not shown). Equipment was selected from industrial suppliers on the basis of its capacity and specifications (e.g., type of material, capacity, and power consumption), and information on their power consumption was used for energy cost estimation. We did not consider the costs of equipment acquisition.

### 2.7. Statistical Analysis

The obtained results were analyzed with TIBCO Data Science Workbench (Statistica) v 14.0.0.15 using one-way analysis of variance (ANOVA) with the Tukey HSD post hoc test. The differences were considered significant at a level of 95% (*p* < 0.05).

## 3. Results

### 3.1. SCBA Characterization

**Figure 1:**
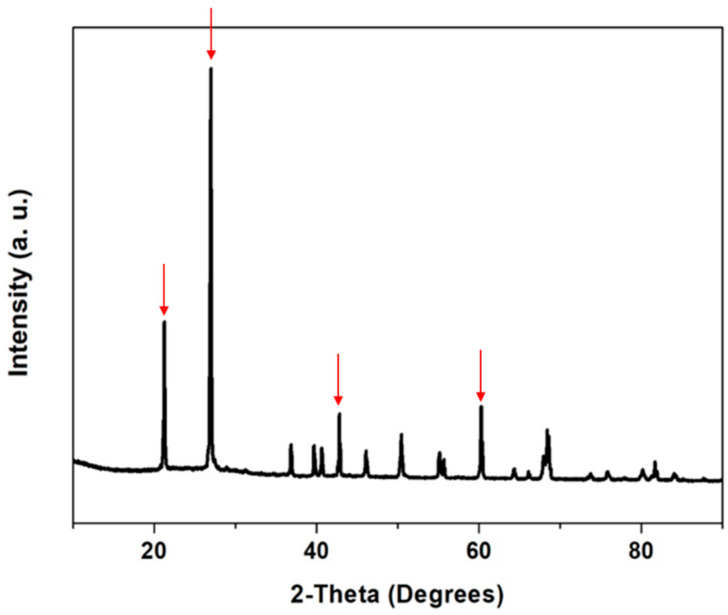
Powder X-ray Diffraction (PXRD) analysis of the sugarcane bagasse ash.

Sugarcane bagasse ashes presented 5.58 ± 0.05 g/100 g of moisture content, 82.28 ± 0.37 g/100 g of minerals, and 12.14 ± 0.16 g/100 g of organic matter. The minerals present in SCBA and their concentration are presented in Table 2. Silicon was the most abundant element, followed by potassium, iron, calcium, magnesium, and phosphorous. The concentrations of the trace minerals sodium, aluminum, zinc, boron, copper, nickel, lead, and cadmium did not differ significantly between them (*p* > 0.005). With silica present in significantly higher concentrations than other minerals (*p* < 0.01), SCBA proved to be an efficient source of this mineral. In fact, sugarcane crops can take up between 500 and 700 kg Si.ha^−1^, while rice husk, a more studied silicon source, absorbs between 230 and 470 kg Si.ha^−1^ [20]. Nevertheless, the silica present in SCBA is mainly in crystalline form, as confirmed by the powder X-ray diffraction analysis (Figure 1). The SCBA diffractogram presented sharp reflections at 20.8°, 26.6°, 50.2°, and 59.9°, identified in Figure 1, which are typical of quartz [21]. This crystalline phase of silica in the SCBA is related to the conditions of combustion, mainly time and temperature, which can convert amorphous silica into crystalline form, and to the presence of sand from the soil that adheres to the sugarcane plant and remains in its by-products. Although the initial SCBA sieving step significantly contributed to reducing the process yield (ca. 52% of total ash mass is discarded during this step), it allowed the removal of all the sand and thus guaranteed that the silica produced afterward did not contain any mineral derived silica; therefore, it can be considered biogenic.

### 3.2. Characterization of Biogenic Silica Prototypes

The production of biogenic silica allowed smooth white powders with a purity of ca. 97.3% and similar chemical compositions to be obtained. Minor contaminants included sodium (1.40 ± 0.23 g/100 g), aluminum (0.59 ± 0.02 g/100 g), iron (0.16 ± 0.041 g/100 g), zirconium (801 ± 41 ppm), magnesium (363 ± 16 ppm), chromium (136 ± 22 ppm), manganese (142 ± 5.5 ppm), zinc (83.5 ± 2.6 ppm), phosphorous (54.9 ± 19.9 ppm), copper (21.1 ± 1.2 ppm), barium (21.3 ± 0.2 ppm), and nickel (5.26 ± 1.08 ppm). Some of these impurities have origins in SCBA, such as potassium, calcium, magnesium, and iron, in accordance with the characterization of several authors for other ashes, such as those from sugarcane, rice husks, and bamboo leaves [20,22,23]. Other contaminants, such as aluminum, chromium, zirconium, copper, and nickel, might have originated in the sodium silicate extraction step. In this step, nickel-plated stainless steel crucibles are used, and due to the extremely alkaline pH of the reaction (pH = 14), the metal might be attacked and contaminate the sample. Nevertheless, neither nickel nor chromium concentrations are fully regulated by the FDA or the European Commission, which suggest maximum concentrations of 10 and 50 ppm, respectively. While the concentration of nickel was within these limits, chromium was far above 50 ppm, suggesting the need for an initial acidic pre-cleaning step to remove excess metal. Finally, other impurities can result from sodium hydroxide pellets used in the synthesis of silica [20]. With a potential application in cosmetics formulations, it is also critical to evaluate the presence of heavy metals; arsenic, cadmium, lead, and mercury were present at trace concentrations of 0.190 ± 0.11 ppm, 23.2 ± 7.3 ppb, 1.52 ± 0.51 ppm, and < 50 ppb, respectively. According to the U.S. Food and Drug Administration (FDA), the maximum concentrations allowed for these heavy metals in most cosmetic formulations are 3 ppm for arsenic, 20 ppm for lead, and 1 ppm for mercury, so regarding the absence of heavy metals, biogenic silica is suitable for potential cosmetic applications [24].

The greatest silica yield was obtained for the silica gel process, 198 g silica/kg SCBA, while the precipitated silica microparticles process provided a yield of 178 g silica/kg SCBA. These silica extraction yields were much lower than others reported for similar biomasses, ranging from 45 to 90% for sugarcane or rice husk ashes, mainly due to the high loss of biomass during the sieving process, which allows the production of 100% biogenic silica [9,11,22,25].

#### 3.2.1. Powder X-ray Diffraction

**Figure 2:**
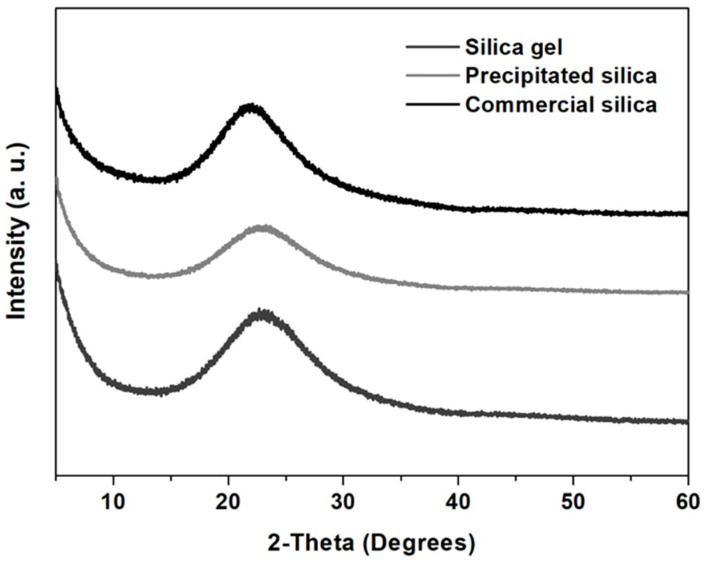
Powder X-ray Diffraction (PXRD) analysis of silica gel, precipitated and commercial silica.

The Powder X-ray diffraction patterns of the precipitated silica gel powder and the commercial silica powder Spheron L-1500, an amorphous silica benchmark widely used in cosmetic formulations, are depicted in Figure 2. In all cases, the diffractograms exhibited a single broad reflection centered at 22.5°, indicating the amorphous nature of these materials. The high purity of the synthesized products can be also corroborated by the absence of additional reflections [20,26].

#### 3.2.2. FT-IR Spectroscopy

**Figure 3:**
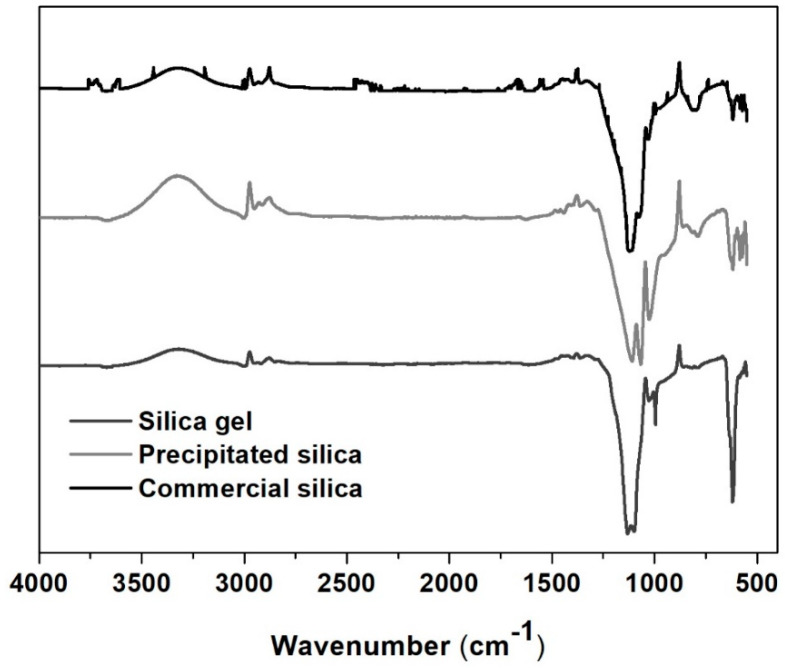
Fourier-Transform Infrared (FT-IR) spectra of silica gel, precipitated and commercial silica powders.

The analysis of the FTIR spectra (Figure 3) revealed broad bands at around 3450 cm^−1^ for both prototypes and the commercial benchmark, corresponding to the overlapping of the O–H stretching bands of hydrogen-bonded water molecules and the SiO-H stretching of hydrogen-bonded surface silanols [27]. It was also possible to observe, for silica gel microparticles, a band at 1650–1500 cm^−1^ corresponding to the deformation vibrations of the adsorbed water, which is common in silica aerogels due to the presence of surface silanol groups and was confirmed by the presence of a sharp peak at 996 cm^−1^, which provided higher hydrophilicity compared with other types of silica [27]. The largest band between 1240–1040 cm^−1^ represented the asymmetric stretching vibrations of siloxane covalent bonds (Si-O-Si), which shifted to 1025 cm^−1^ for precipitated silica, while the peak at 887 cm^−1^ represented the symmetric stretching vibration of the same group, characteristic of the silica network.

#### 3.2.3. Particle Morphology

**Figure 4:**
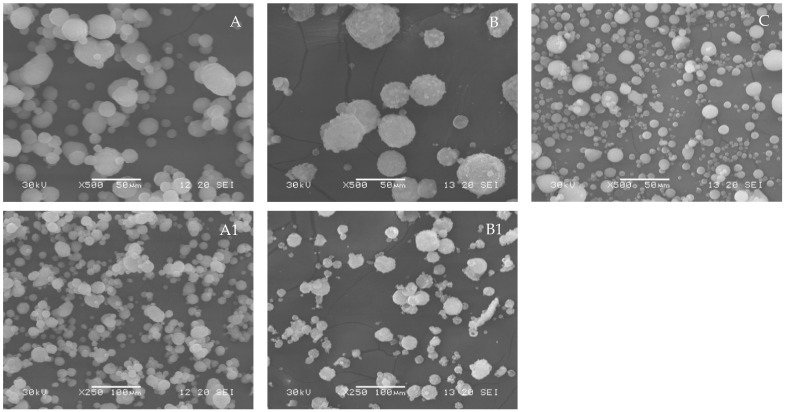
SEM micrographs of (**A**) (X500, 50 μm), (**A1**) (X250, 100 μm) silica gel, (**B**) (X500, 50 μm), (**B1**) (X250, 100 μm) precipitated silica microparticles, and (**C**) benchmark silica Spheron L-1500.

The particle shape is critical to achieving the desired functional performance of silica, as non-spherical silica particles do not allow silky powder to be obtained and significantly increase the oil absorption capacity, leading to a dry sensation when applied to the skin. Scanning Electron Microscope (SEM) images of silica powders demonstrate the clear spherical appearance of silica microparticles produced following both methodologies (Figure 4). Spheron L-1500 showed the presence of individualized spherical particles with low surface rugosity. The microstructure of precipitated silica showed the presence of spherical particles with a smooth surface without the presence of perceptible pores, although some of the particles formed small agglomerates. The microstructure of silica gel also revealed the presence of microspheres without significant agglomerates but with particles that presented higher surface porosity compared with the benchmark or precipitated silica.

Besides the particle shape, the size of silica particles also affects their technical performance, including the oil and water absorption capacity. Table 3 presents the particle size distribution of precipitated silica, silica gel, and commercial benchmark particles. The particle size distribution of precipitated silica microparticles ranged from 1 to 35 μm, with an average size of around 6.9 μm and a span diameter of 1.17, suggesting a narrow range of particle sizes. Silica gel presented a lower dispersion of particle sizes, ranging from 1 to 20 μm but a higher span of 1.46. Both prototypes presented similar particle size distributions to the benchmark Spheron L-1500, which presented particle sizes ranging from 2 to 25 μm. The wider particle size distribution in the precipitated silica can be explained by the drying process at 105 °C, which promoted the formation of smaller spherical particles (200–500 nm) that are later aggregated into larger particles through atomization. Depending on the dispersion of the silica suspension before the atomization and the drying conditions, specifically the sample flow and aspiration rate, the aggregation can result in microparticles with a wide range of sizes. Kortesuo and co-workers also used a spray dryer to encapsulate drugs into silica gel microparticles, achieving similar particle sizes ranging from 1 to 40 μm [28].

With both methods, it was possible to obtain a silica powder without the presence of nanoparticles (<1000 nm), ensuring that it was suitable for cosmetic application. According to Scientific Committee on Consumer Safety (SCCS) guidance on nanomaterials in cosmetics, there is a concern related to the use of nanosilica in cosmetic formulations, as they might be able to permeate the skin barrier and be carried by the bloodstream and accumulate in the internal organs, which could potentially lead to toxicity [29].

### 3.3. Oil and Water Absorption Capacities

**Figure 5:**
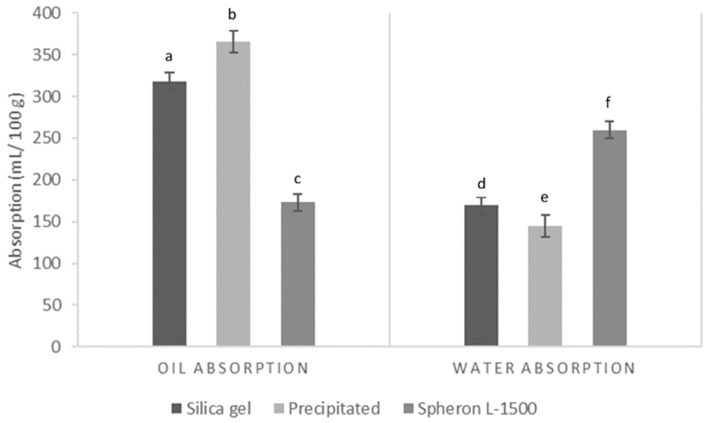
Oil and water absorption capacity of silica gel and precipitated silica microparticles. Results are expressed as average ± SD (n = 3) and different letters (a–f) represent statistical differences.

Undesirable skin shine is composed of sebum, sweat, dead skin cells, and environmental impurities. Thus, an ingredient that presents both humidity and the ability to absorb sweat and oil and remain dry after being absorbed, will have a better mattifying effect than ingredients that mainly absorb oil or that become wet and cakey after absorbing moisture [30]. Spherical silica particles are frequently used in cosmetics due to their great capacity to absorb sweat and oil, leading to a decrease in light reflection and improvement of soft focus and mattifying effect. The oil and water absorption capacities of silica microparticles are presented in Figure 5. Silica gel microparticles presented a significantly lower (*p* < 0.05) oil absorption capacity, 289.7 ± 23.4 g sunflower oil/100 g silica, than precipitated silica, which presented an oil absorption of 332.9 ± 11.7 g sunflower oil/100 g silica. Both synthesized prototypes presented an oil absorption capacity significantly higher than the cosmetic benchmark Spheron L-1500 (*p* < 0.01). These results could be explained by the lower interparticle porosity (0.159) of Spheron L-1500 compared with silica gel, with an interparticle porosity of 0.448, and compared with precipitated silica, with an even higher interparticle porosity of 1.120 (data not shown).

These results are in accordance with several authors, who achieved oil absorption values between 150 and 380 g oil/100 g silica aerogel [31,32,33] and between 250 and 500 g oil/100 g for precipitated silicas [34,35]. Regarding the water absorption capacity, the opposite effect was observed, with silica gel presenting a significantly higher (*p* < 0.05) water absorption capacity, of 169.4 ± 10.4 mL/100 g, while precipitated silica presented a water absorption of 145.0 ± 7.1 g/100 g. By contrast, both sugarcane-derived silica prototypes presented a significantly lower (*p* < 0.01) water absorption capacity than Spheron L-1500, which was 262.0 ± 7.5. These water absorption values are, however, far higher than the water absorption reported by Chindaprasirt and Rattanasak [10]. These results confirm the high specific surface area of silica gel (639 m^2^/kg, data not shown), which is a well-known moisture absorber widely used in different products and applications as a desiccant. Amorphous silica comprises an inorganic network of SiO_2_ units bonded through hydrophobic siloxane groups and holding hydrophilic surface silanol (Si-OH) groups [30].

### 3.4. Production Cost Estimation

**Figure 6:**
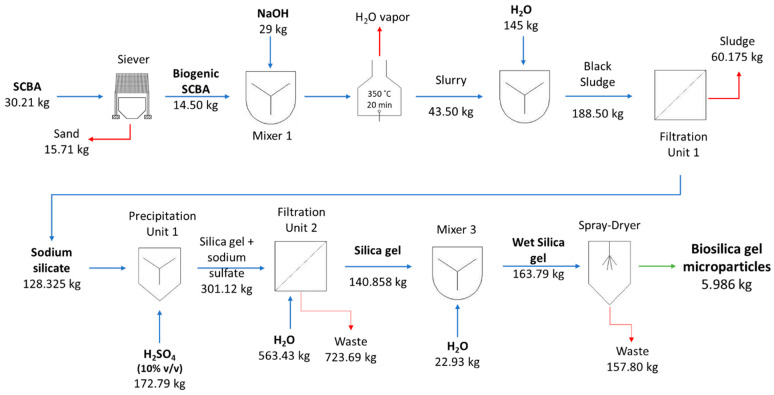
Mass balance, key unit operations, and process inputs in the production of silica gel microparticles.

**Figure 7:**
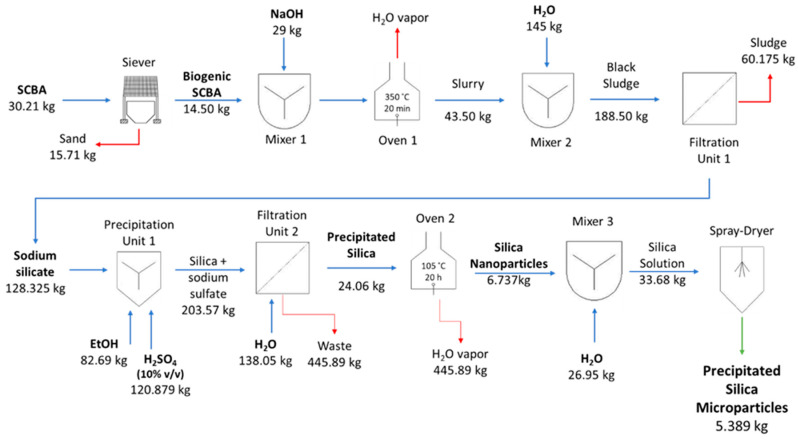
Mass balance, key unit operations, and process inputs in the production of precipitated silica microparticles.

A comprehensive analysis of economic viability is needed to predict the feasibility of large-scale production of biogenic silica; therefore, both lab processes’ mass balances, presented in Figure 6 and Figure 7, were assessed to estimate the costs of large-scale production. Table 4 presents the overall estimated production costs of both silica prototypes, which were EUR 10.89/kg and EUR 28.95/kg for silica gel and precipitated silica microparticles, respectively. The biggest expense was the overhead costs, ca. 44.4% of the total cost for both prototypes, which was empirically defined as 80% of the chemical and energy costs. Although the overhead percentage value applied for both processes was similar, the absolute costs were very different: EUR 4.84/kg for silica gel and EUR 12.86/kg for precipitated silica (2.7-fold higher).

This difference is explained by the higher consumption of chemicals in the production of precipitated silica, in particular the use of absolute ethanol, which represented an increase in chemical costs, from 29% (EUR 3.18/kg) for silica gel to almost 36% (EUR 10.30/kg) in precipitated silica. Regarding the energy costs, they were also expected to be higher in precipitated silica, as this process requires an additional operation unit—an oven. Thus, the total energy cost for silica gel was EUR 2.87/kg, with the heating reactor and spray-dryer units being the most expensive steps, while the energy cost for precipitated silica was EUR 5.79/kg, with the oven step portraying 45% of energy costs.

To make this economic simulation as realistic as possible, the costs for chemicals and energy were based on bulk quantity prices, although the mass balances were determined at the lab scale. Lab scale processes are usually more prone to lower yields, whether by the mass losses along the process or the reduced yield of some lab equipment, such as the spray dryer.

## 4. Conclusions

Two prototypes of biogenic amorphous silica were synthesized from sugarcane bagasse ashes— precipitated and silica gel microparticles—with a purity of up to 98%. Their microstructure was composed of mesoporous spherical particles with sizes between 1 and 15 μm, providing optimal oil and moisture absorption capacities. Associated with the lack of nanometric particles and heavy and potential sensitizer metals, these prototypes are safe and ideal for cosmetic applications. Moreover, a process development study and cost analysis allowed the optimization of the process, guaranteeing the economic viability of silica as a commercial ingredient. These prototypes represent a breakthrough in the cosmetic market that lacks sustainable and biogenic options.

## Figures and Tables

**Table 1:** Key assumptions used to design the production process of biogenic silica.

Energy price (EUR/kW) Water (EUR/kg)	0.14 ^1^
	0.002 ^2^
Sodium hydroxide (EUR/kg) Absolute ethanol (EUR/kg) Sulfuric acid (EUR/kg)	0.34 0.48 0.25
Sugarcane ash cost	Not considered
Overheads	80% of chemicals and energy costs

^1^ Eurostat, statistics on electricity price [19]; ^2^ local price.

**Table 2:** Elements present in sugarcane bagasse ash.

Element	Concentration (g.100 g^−1^)	Element	Concentration (ppm)
Silicon	70.26 ± 0.03	Manganese	843.7 ± 2.38
Potassium	7.23 ± 0.023	Sodium	248 ± 0.7
Iron	1.72 ± 0.005	Aluminum	121.0 ± 0.34
Calcium	1.64 ± 0.005	Zinc	94.98 ± 0.27
Magnesium	0.83 ± 0.002	Boron	50.65 ± 0.14
Phosphorous	0.44 ± 0.001	Copper	44.44 ± 0.13
		Nickel	7.8 ± 0.02
		Lead	3.54 ± 0.01
		Cadmium	2.29 ± 0.01

**Table 3:** Particle size distribution of silica microparticles.

	D_10_	D_50_	D_90_	Span
Silica gel	2.4 μm	3.9 μm	8.1 μm	1.46
Precipitated silica	4.9 μm	6.9 μm	13.0 μm	1.17
Spheron L-1500	3.1 μm	4.8 μm	9.3 μm	1.29

D_10_, D_50_, and D_90_—size at which 10, 50, and 90% of the particles are under.

**Table 4:** Comparison of the estimated production cost of silica gel and precipitated silica in terms of chemicals, energy, and overhead.

	Silica Gel (EUR/kg Silica)	Precipitated Silica (EUR/kg Silica)
**Chemicals** Sodium Hydroxide	**3.18** 1.65	**10.30** 1.83
Sulfuric acid	1.25	0.95
Absolute ethanol	-	7.36
Water	0.29	0.13
**Energy** Siever Mixer 1 + Oven 1 + Mixer 2	**2.87** 0.002 1.27	**5.79** 0.002 1.41
Filtration Unit 1 Precipitation Unit 1 Filtration Unit 2 Oven Mixer 3 Spray-Dryer	0.05 0.13 0.09 - 0.04 1.30	
**Overheads**	4.84	12.86
**Total Production Cost**	**10.89**	**28.95**

## Data Availability

Not applicable.
